# Neural and psychological correlates of post-traumatic stress symptoms in a community adult sample

**DOI:** 10.1093/cercor/bhae214

**Published:** 2024-05-30

**Authors:** Sierra A Bainter, Zachary T Goodman, Lauren B Kupis, Kiara R Timpano, Lucina Q Uddin

**Affiliations:** Department of Psychology, University of Miami, 5665 Ponce de Leon Blvd, Coral Gables, FL 33146, United States; Department of Psychology, University of Miami, 5665 Ponce de Leon Blvd, Coral Gables, FL 33146, United States; Department of Psychiatry and Biobehavioral Sciences, University of California Los Angeles, 760 Westwood Plaza, Los Angeles, CA 90095, United States; Department of Psychology, University of Miami, 5665 Ponce de Leon Blvd, Coral Gables, FL 33146, United States; Department of Psychiatry and Biobehavioral Sciences, University of California Los Angeles, 760 Westwood Plaza, Los Angeles, CA 90095, United States; Department of Psychology, University of California Los Angeles, 1285 Psychology Building, Box 951563, Los Angeles, CA 90095-1563, United States

**Keywords:** brain networks, resting state fMRI, PTSD, SSVS, stochastic search variable selection

## Abstract

A multitude of factors are associated with the symptoms of post-traumatic stress disorder. However, establishing which predictors are most strongly associated with post-traumatic stress disorder symptoms is complicated because few studies are able to consider multiple factors simultaneously across the biopsychosocial domains that are implicated by existing theoretical models. Further, post-traumatic stress disorder is heterogeneous, and studies using case-control designs may obscure which factors relate uniquely to symptom dimensions. Here we used Bayesian variable selection to identify the most important predictors for overall post-traumatic stress disorder symptoms and individual symptom dimensions in a community sample of 569 adults (18 to 85 yr of age). Candidate predictors were selected from previously established risk factors relevant for post-traumatic stress disorder and included psychological measures, behavioral measures, and resting state functional connectivity among brain regions. In a follow-up analysis, we compared results controlling for current depression symptoms in order to examine specificity. Poor sleep quality and dimensions of temperament and impulsivity were consistently associated with greater post-traumatic stress disorder symptom severity. In addition to self-report measures, brain functional connectivity among regions commonly ascribed to the default mode network, central executive network, and salience network explained the unique variability of post-traumatic stress disorder symptoms. This study demonstrates the unique contributions of psychological measures and neural substrates to post-traumatic stress disorder symptoms.

## Introduction

Approximately 90% of adults in the United States report exposure to a traumatic event, and yet only 10% of the population meets lifetime criteria for DSM-5 post-traumatic stress disorder (PTSD) (Kilpatrick et al. 2013). PTSD is characterized by re-experiencing phenomena, avoidance, negative alterations in cognition or mood, and hyperarousal symptoms (American Psychiatric Association 2013). Re-experiencing symptoms include intrusive thoughts, flashbacks, and nightmares. Individuals with PTSD may avoid these intrusive thoughts, situations, and reminders of the traumatic event and exhibit hyperarousal, which can include elevated threat sensitivity, exaggerated startle, and hypervigilance. Negative alterations in cognition or mood may include an inability to remember important aspects of the trauma, feelings of detachment, diminished interest, or anhedonia.

Various factors—including personality, cognitive, and behavioral characteristics—may contribute to the development and persistence of PTSD. Personality features, including negative affectivity and neuroticism, are also related to the risk, expression, or prognosis of trauma-related psychopathology (Zuckerman 1999). PTSD is further characterized by behavioral dysregulation, including poor or atypical sleep (Pillar et al. 2000), increased dependency on alcohol, tobacco, or other drugs (Vandrey et al. 2014), and diminished engagement in physical activity (Nilsson et al. 2021). Impulsivity and cognitive processes related to difficulties with emotional control have also been associated with elevated PTSD symptoms in cross-sectional studies (Tull et al. 2007). Multiple facets of impulsivity are seen as core factors mediating exposure to trauma and the development of greater PTSD symptom severity (Contractor et al. 2018). In addition to basic impulsivity driven by a lack of perseverance or follow-through, emotion-related impulsivity has also been linked with PTSD, particularly with respect to reacting rashly in response to negative emotions (Ceschi et al. 2014). Finally, cognitive processes implicated in PTSD include rumination, thought suppression, and experiential avoidance (LoSavio et al. 2017).

In parallel to the above literatures, neuroimaging studies have examined the neural substrates of PTSD pathophysiology. An influential neurocircuitry model originally proposed that PTSD results from the combination of: (i) hyperresponsivity of the amygdala related to hyperarousal symptoms, (ii) abnormal functioning of the hippocampus underlying PTSD-related differences in learning and memory, and (iii) hypoactivation of prefrontal cortical brain regions responsible for top-down inhibitory control processes [including the anterior cingulate cortex (ACC), ventromedial prefrontal cortex, subcallosal cortex, and orbitofrontal cortex] (Rauch et al. 2006). Recent studies have also implicated atypical connectivity within and between several core intrinsic connectivity networks (ICNs), including the default mode network (DMN), salience network (SN), and central executive network (CEN). The SN plays an important role in the attentional capture of relevant stimuli (Uddin 2015). The CEN is important for maintaining and manipulating information in working memory, and the DMN is involved in self-referential mental processes (Menon 2011). A recent review (Akiki et al. 2017) and meta-analysis (Bao et al. 2021) summarize that PTSD may be characterized primarily by hypoactivity and hypoconnectivity in the DMN and CEN, along with overactivity and hyperconnectivity in the SN. Importantly, patterns of both increased and decreased connectivity have emerged, which may depend on study differences relating to subjects (age, trauma type, and age of occurrence) and fMRI acquisition and analysis approaches (Sripada et al. 2012; Breukelaar et al. 2021).

Establishing which predictors are most strongly associated with PTSD symptoms remains challenging. A key limitation of the existing research on factors related to PTSD is that few studies have adequate power or statistical methods to consider a sizeable number of factors simultaneously within or across domains (Tortella-Feliu et al. 2019). Even large samples are insufficiently powered using traditional approaches to simultaneously consider multiple correlated factors and adequately adjust for the large number of comparisons (Swartz et al. 2008). This is because with multiple correlated predictors, the strength of predictors changes depending on which other predictors are controlled in the model (Viallefont et al. 2001), and as a result, it is unclear which factors robustly predict outcomes. Further, research on psychological factors and neural substrates of trauma-related psychopathology is usually conducted in isolation. An important and outstanding question is: when considering multiple factors across domains, which measures are the most important predictors of PTSD symptoms? Differences in brain network connectivity play an important role in the affective and cognitive symptoms of several psychiatric disorders; however, it is not known whether brain network and psychological measures explain overlapping or distinct variability in symptoms.

Substantial comorbidity between PTSD and other psychiatric disorders, especially depressive disorders, is an additional challenge to understanding which factors may be uniquely related to PTSD symptoms. Studies have found that the majority of individuals with PTSD meet criteria for at least one other psychiatric disorder, with major depression being the most common comorbidity, followed by substance use disorders and other anxiety disorders (Brady et al. 2000). The question of comorbidity with PTSD is complex and raises important issues related to diagnostic criteria and treatment (Brady et al. 2000; Pai et al. 2017).

Risk and vulnerability research on PTSD is further complicated by the fact that it is a highly heterogeneous disorder (Pietrzak et al. 2014). Despite heterogeneity in symptom patterns and severity, research identifying risk factors and correlates of PTSD often uses case-control designs that have 2 important limitations. First, current initiatives, such as the National Institute of Mental Health’s Research Domain Criteria (RDoC), highlight the importance of considering psychiatric symptoms dimensionally, including sub-clinical symptom presentations (Insel et al. 2010). Second, it is important to understand how specific factors may be differentially associated with different symptom domains (Minihan et al. 2018).

We examined the relationships between psychological and brain functional connectivity (FC) measures and PTSD symptom domains in a large (*n* = 569), community cross-sectional sample of adults from the Nathan Kline Institute’s publicly available database (Nooner et al. 2012). We used a dimensional approach to capture PTSD symptoms, given the findings that PTSD captures the upper end of a broad stress-response continuum (Ruscio et al. 2002) and increasing calls for using a dimension-based approach to more reliably and validly capture clinical constructs (Kraemer et al. 2004). We considered the range of assessments available in the NKI study and included behavioral measures that had robust and consistent support for a relationship with PTSD in past research. Specifically, we selected the transdiagnostic psychological measures of impulsivity and personality traits (Contractor et al. 2018), alongside measures of behavioral dysregulation, such as sleep quality and substance use. The brain regions included were selected based on meta-analytic findings of atypical FC in individuals with PTSD relative to trauma-exposed controls (Patel et al. 2012). Our primary aim was to consider models for each of the symptom domains, in which we simultaneously considered many theoretically relevant predictors from both psychological and neural domains using a Bayesian variable selection method. Our second aim was to consider follow-up analyses including depression symptoms as a candidate predictor in order to understand which factors uniquely relate to PTSD symptoms while controlling for this important psychiatric comorbidity (Brady et al. 2000; Armenta et al. 2019).

## Materials and methods

### Participants

A sample of 670 unrelated adults was drawn from the Enhanced Nathan Kline Institute’s Rockland Sample (NKI-RS) (Nooner et al. 2012), a community sample of participants across the lifespan. Though the ethnic and socioeconomic demographics of Rockland County, New York, closely resemble those of the United States, and the study aimed to balance recruitment across zip codes, age, sex, and ethnicity, the sample is not formally representative. For example, while Rockland County is 51% female, the obtained NKI sample was 59.4% female. Participants completed multiple self-report measures, cognitive tasks, a structured clinical interview, and a resting state fMRI scan over 1 or 2 d. Each participant’s scan was evaluated by trained researchers for imaging artifacts, and subjects were excluded for average head motion exceeding a framewise displacement of 0.50 mm (Power et al. 2012).

The final sample passing this quality control procedure and with at least partial self-report data consisted of 569 participants (63% female) ranging from 18 to 85 yr of age (*M* = 43.5, *SD* = 18.4). This sample was composed of participants from diverse racial backgrounds, with the majority identifying as White (72%). Other racial groups represented in the sample included Black (18%), Asian (6%), and those identifying as belonging to other racial groups (4%). Additionally, 13% of the sample identified as Hispanic or Latino. The sample of participants for whom data were excluded were older on average than the included sample (*M* = 48.5) but were not significantly different in terms of gender, race, or ethnicity.

### Image Acquisition and Preprocessing

Participants completed a 10-min multiband (x4) EPI resting-state fMRI scan collected on a Siemens Trio 3.0 T scanner with a T_1_ anatomical image (2^3^mm, 40 interleaved slices, TR = 1.40s, TE = 30 ms, flip angle = 65°, FOV = 224 mm, 404 volumes). Participants were instructed to keep their eyes open and fixate on a cross centered on the screen (https://fcon_1000.projects.nitrc.org/indi/enhanced/mri_protocol.html).

Resting state fMRI data were preprocessed using FSL, AFNI, and SPM functions through DPARSF-A in DPABI (Yan et al. 2016). The first 5 images were removed to allow the MRI signal to reach equilibrium. Data were despiked using AFNI 3dDespike, realigned and normalized with DPARSF-A into 3-mm MNI space, and then smoothed to 6 mm with AFNI 3dBlur. The ICA-FIX classifier was trained on hand-classified independent components separated into noise and non-noise categories on the data from 24 subjects randomly chosen across the lifespan (Griffanti et al. 2014; Salimi-Khorshidi et al. 2014). The component classifications were then fed into FMIRB’s ICA-FIX classification algorithm (Griffanti et al. 2014) to classify noise and non-noise components from individual subject data before conducting nuisance regression of classified noise components in MNI space. Next, 24 motion parameters (Friston et al. 1996) and linear trends were regressed out before application of a band-pass filter (0.01 to 0.10 Hz).

### Parcellation

The Human Brainnetome Atlas (Fan et al. 2016) was used to derive a total of 246 regions of interests (ROIs), which include 210 cortical and 36 subcortical regions. Of these, 106 ROIs were selected for inclusion in this study based on regions identified in a quantitative meta-analysis of whole-brain functional neuroimaging investigations of PTSD (Patel et al. 2012). Importantly, the meta-analysis synthesized results based on a range of experimental paradigms as well as resting-state conditions. We included ROIs that corresponded to the regions identified as having altered activation in PTSD relative to trauma-exposed controls (see Table 1).

**Table 1:** From Patel et al. (2012) meta-analysis to identify regions with altered activity in PTSD relative to trauma-exposed controls.

**Side**	**Region (from** Patel et al. 2012**)**	**Abbr.**	**Brainnetome Atlas labels**
PTSD > Controls (Table 3b)			
R	Cingulate gyrus^a^	rCG	176,178,180,182,184,186,188
L	Insula	lIns	163,165,167,169,171,173
R	Precuneus	rPreC	148,150,152,154
L	Superior temporal gyrus	lSTG	69,71,73,75,77,79
L	Inferior temporal gyrus	lITG	89,91,93,95,97,99,101
L	Fusiform gyrus	lFG	103,105,107
R	Thalamus	rThal	232,234,236,238,240,242,244,246
PTSD < Controls (Table 4b)			
R	Cingulate gyrus^a^	rCG	*Included above*
R	Orbitofrontal CORTEX	rOFC	42,44,46,48,50,52
R	Inferior frontal gyrus	rIFG	30,32,34,36,38,40
R	Medial frontal gyrus (superior frontal gyrus)	rSFG	2,4,6,8,10,12,14
L	Frontal pole (orbitofrontal gyrus)	lOFG	41,43,45,47,49,51
R	Precentral gyrus	rPCG	54,56,58,60,62,64
R	Middle frontal gyrus	rMFG	16,18,20,22,24,26,28
L	Parahippocampal gyrus	lPHG	109,111,113,115,117,119
L	Thalamus	lThal	231,233,235,237,239,241,243,245
L	Inferior frontal gyrus	lIFG	29,31,33,35,37,39
L	Insula	lIns	*Included above*
L	Middle frontal gyrus	lMFG	15,17,19,21,23,25,27

*Note.* Atlas labels refer to the region as represented by Brainnetome atlas.

^a^Region from Patel et al. was labeled Dorsal Anterior Cingulate and has no exact corresponding region in the Brainnetome partioning, here we substitute the Cingulate Gyrus.

This choice of control group comparison is relevant because some regions, such as the amygdala and hippocampus, are identified only when comparing PTSD to non-trauma-exposed controls, which suggests that alterations in these regions may be related to trauma exposure generally rather than PTSD symptoms specifically (Patel et al. 2012). The regions identified from the meta-analysis included regions from the SN, CEN, and DMN, the left insula, left fusiform gyrus, and right precentral gyrus, and 2 regions also identified in the traditional neurocircuitry model of PTSD (Rauch et al. 2006). Individual-level FC matrices were generated by correlating the time series of each ROI with every other ROI time series. The Fisher-transformed correlation coefficients were used in subsequent analyses.

### Self-report measures

#### PTSD symptoms

The UCLA PTSD Reaction Index for DSM-IV (Steinberg et al. 2004) is a 48-item self-report used to screen for exposure to traumatic events and subsequent post-traumatic symptoms. Originally intended for use with adolescents, the measure has been used with both adolescents and adults to facilitate comparisons of PTSD symptoms across development since neither diagnostic criteria nor the reading level of the instrument differs across age groups (Allwood 2019). The measure first screens for a history of a traumatic event and then yields an overall severity score of associated PTSD symptoms (range: 0 to 17), along with subscale scores of intrusion (range: 0 to 20), avoidance/numbing (range: 0 to 28), and arousal (range: 0 to 20), which map onto DSM-IV criteria.

#### Personality and impulsivity measures

The Adult Temperament Questionnaire (ATQ) short form is a 77-item self-report questionnaire assessing personality dimensions of effortful control, negative affect, extraversion/surgency, and orienting sensitivity (Evans and Rothbart 2007). Negative affectivity is a prospective predictor of PTSD, as is neuroticism (Rademaker et al. 2011).

The Urgency, Premeditation, Perseverance, Sensation Seeking Impulsive Behavior Scale (UPPS-P) is a 59-item self-report that assesses 5 subscales (negative urgency, lack of premeditation, lack of perseverance, sensation seeking, and positive urgency) that are used to measure dimensions of impulsivity in adolescents and adults (Cyders et al. 2007). Negative and positive urgency reflect the tendency to act rashly in response to negative and positive emotions, respectively. Each aspect of impulsivity plays a different role in explaining risky behaviors (Whiteside and Lynam 2001; Cyders et al. 2007).

#### Health behavior measures

The Pittsburgh Sleep Quality Index (PSQI) is a 10-item measure of sleep quality (Buysse et al. 1989). Sleep dysregulation is commonly associated with post-traumatic symptoms (Pillar et al. 2000), and the PSQI has demonstrated sensitivity to sleep dysregulation in those with PTSD (Biggs et al. 2020). Total physical activity was measured using the International Physical Activity Questionnaire (IPAQ) (Booth 2000). The IPAQ asks about time spent being physically active in the last 7 d. The Fagerström Test for Nicotine Dependence (FTND) is a screening instrument that assesses nicotine dependence in adults who are currently smokers or have been in the past 2 yr (Heatherton et al. 1991). Alcohol use was measured as the number of days in which alcohol was consumed in the past month, extracted from the Comprehensive Addiction Severity Index for Alcohol (CASI-A) (Meyers et al. 1995).

#### Depression symptoms

The Beck Depression Inventory (BDI-II) is a 21-item self-report questionnaire assessing the current severity of depression symptoms (Beck et al. 1996). Questions assess prototypical depressive symptoms over the most recent 2 wk, and the measure demonstrates strong psychometric properties across clinical and non-clinical populations (Wang and Gorenstein 2013).

### Statistical analyses

In total, 122 candidate predictors were included to predict the severity of overall PTSD symptoms and the three PTSD subscales. The number of cases available for each self-report measure is shown in Table 2. Missing data rates for self-report measures ranged from 0 to 18% (average = 2.2%), with 425 participants having complete data for all measures. Most missingness was by design: adults over 65 (*n* = 97) were not administered the BDI-II to measure depression but had available data on all other measures. To retain a large, lifespan adult sample, we used Blimp software 3.0.65 (Keller and Enders 2019) to generate *m* = 50 imputations of missing values for the self-report measures using fully conditional specification.

**Table 2:** Demographic characteristics and descriptive statistics for self-report measures.

		**N**	**M**	**SD**	**Min**	**Max**
	Age	569	43.5	18.44	18	85
PTSD symptoms (UCLA PTSD index)	Overall severity	562	2.98	3.02	0	13
	Re-experiencing	562	1.17	2.37	0	14
	Avoidance	562	1.94	3.83	0	26
	Arousal	562	2.5	3.56	0	16
Personality/temperament (ATQ)	Negative affect	561	3.65	0.7	1.96	5.65
	Effortful control	561	4.88	0.73	2.58	6.95
	Extraversion/surgency	561	4.35	0.69	2.06	6.35
	Orienting sensitivity	561	4.49	0.81	2.2	6.6
Impulsivity (UPPS)	Negative urgency	563	23.68	6.58	12	46
	Lack of premeditation	563	19.56	4.54	11	35
	Lack of perseverance	563	17.82	4.39	10	32
	Sensation seeking	563	29.4	8.03	12	48
	Positive urgency	563	21.59	7.28	14	52
Behavioral dysregulation	Poor sleep quality (PSQI)	561	4.75	3.15	0	17
	Physical sctivity (IPAQ)	567	2371.7	6532.7	0	55471.5
	Nicotine sependence (FTND)	567	0.29	1	0	7
	Alcohol use (CASI)	529	4.27	6.25	0	31
Psychiatric comorbidity	Depression (BDI)	467	6.23	6.96	0	34

Stochastic search variable selection (SSVS) was used to identify the most important predictors of PTSD symptoms among the set of candidate neural and self-report measures. SSVS is a Bayesian variable selection approach that can be used to estimate the marginal inclusion probability (MIP) for each predictor; these are the probabilities that each predictor should be included to predict the outcome, controlling for uncertainty in which other predictors are included in the model (Bainter et al. 2020). By accounting for model uncertainty, Bayesian variable selection methods both increase power and protect against false-positive results, compared with traditional approaches (Viallefont et al. 2001; Swartz et al. 2008). We used the SSVS R package for analyses using the default prior inclusion probability of 0.5 for each predictor and the default of 20,000 iterations, discarding 5,000 as burn-in (Bainter et al. 2022). To allow the prior to uniformly influence all predictors, the predictors are standardized. The model-averaged regression coefficients are also produced (averaging over both zero and non-zero estimates) and are comparable as standardized with respect to the predictors but not the outcome.

We compared results for overall PTSD symptoms scores and for the subdimensions of re-experiencing, arousal, and avoidance symptoms. Because of the high rates of comorbidity between PTSD and depression, we examined results both excluding and controlling for depression. The pattern of MIPs averaged across imputations was examined graphically to determine meaningful cutoffs for each outcome. Bayesian variable selection can be combined with MI in several ways. The approach used here is described as “impute, then select” (Yang et al. 2005) and is straightforward to apply using standard MI software.

## Results

Of the sample of 380 participants, 67.6% (*n* = 7 missing) reported exposure to a traumatic event, similar to prevalence estimates of 70% of the world’s adult population (Kessler et al. 2017). Of participants who reported a traumatic event, most reported events that had occurred 3 or more years in the past (*n* = 275) or between 1 and 3 yr prior (*n* = 56). Twenty-one participants reported a traumatic event that had occurred between 7 and 12 mo prior (*n* = 21), and <20 reported a traumatic event in the past month (*n* = 5) or past 2 to 6 mo (*n* = 14). There was not a significant relationship between overall symptoms and how long prior the event had occurred *t*(369) = −0.53, *P* = 0.60. The DSM-IV PTSD criteria require that the event involve both actual or threatened death or serious injury, or a threat to the physical integrity of self or others (criterion A1), and a response involving intense fear, helplessness, or horror (criterion A2). A subset of 266 participants (47.3%, *n* = 7 missing) met both the A1 and A2 DSM-IV PTSD criteria (American Psychiatric Association 1994), as captured by the UCLA PTSD Index. Means and standard deviations for the self-reported variables, along with the demographic characteristics of the sample, are included in Table 2. The mean overall PTSD symptom severity was 2.98 (range: 0 to 13).

### Overall PTSD symptom severity

Results for each outcome are shown in Table 3, including MIPs for each selected predictor and model-averaged regression coefficients, both averaged across imputations. For overall PTSD symptom severity, 5 self-report measures and 1 brain connectivity measure were selected. The predictor with the highest MIP was poor sleep quality, which on average (across models) was associated with a 0.46 unit increase in PTSD symptoms per SD increase in poor sleep quality. Of the psychological predictors, two dimensions of impulsivity and one dimension of temperament were selected. Negative urgency predicted higher overall symptoms, while a higher lack of perseverance predicted lower symptom severity. Orienting sensitivity was positively associated with overall symptoms. Nicotine dependence was also associated with symptoms.

**Table 3:** Results from SSVS indicating predictors selected to model each PTSD score.

**Selected predictor**	Outcome			
	**Overall**	**Re-experiencing**	**Avoidance**	**Arousal**
Self-report measures	MIP, Avg *B*	MIP, Avg *B*	MIP, Avg *B*	MIP, Avg *B*
Negative urgency	0.78, 0.36		0.99, 0.89	0.82, 0.48
Lack of perseverance	0.80, −0.37			
Positive urgency		0.99, 0.52		
Negative affect		0.91, 0.36		0.87, 0.51
Extraversion/surgency			0.48, −0.20^a^	
Orienting sensitivity	0.83, 0.33	0.57, 0.16	1.0, 0.79	0.89, 0.44
Poor sleep quality	0.97, 0.46	0.60, 0.17	0.98, 0.58	1.0, 0.76
Nicotine dependence	0.91, 0.39	0.97, 0.35		
Alcohol use				0.77, −0.34
Gender (Ref = Male)			0.82, −0.41	0.54, −0.22
Age		0.52, −0.15		
Brain connectivity measures				
L Middle frontal gyrus– L Inferior frontal gyrus	0.57, −0.31	0.51, −0.25		
L Middle frontal gyrus– R Orbitofrontal cortex		0.59, −0.26		
R Cingulate gyrus– L Parahippocampal gyrus		0.49, 0.22^a^	0.53, 0.38	

*Note.* Results are averaged over 50 imputations. Predictors not selected for any outcome are omitted. Blank cells indicate predictors not selected for a given outcome. ^a^Coefficient was just below the MIP threshold of 0.5.

**Fig. 1:**
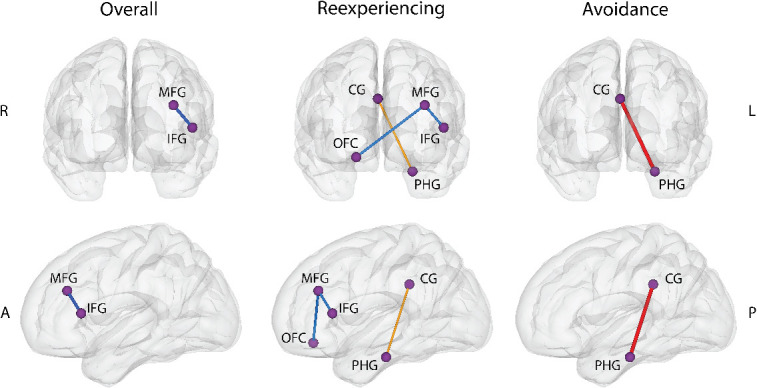
Depiction of selected brain functional connectivity measures for overall, re-experiencing, and avoidance symptoms. Lines connecting regions are weighted by the model-averaged regression coefficients. Blue indicates stronger connectivity is related to lower symptom severity, red indicates greater connectivity is related to higher symptom severity. One connection is shown with yellow, denoting a subthreshold connection.

Of the brain FC measures, only hypoconnectivity between the left inferior frontal gyrus (lIFG) and the left middle frontal gyrus (lMFG) was associated with overall PTSD symptoms. Results for selected brain FC measures are visualized in Fig. 1.

### Subscales

For each of the symptom subscales as for the overall score, poor sleep quality was consistently associated with greater symptom severity. Of the temperament measures, orienting sensitivity was consistently selected as a predictor for each symptom subscale as for the overall score. Higher levels of negative affect also predicted greater re-experiencing and arousal symptoms. Greater negative urgency predicted higher avoidance and arousal symptoms, while positive urgency was selected as a unique predictor for re-experiencing symptoms.

Nicotine dependence, as in the model for overall symptom severity, was a selected predictor related to re-experiencing but not avoidance or arousal. Gender was selected as a predictor associated with avoidance and arousal, with females in the sample predicted to have lower symptoms. Age was selected only in the model for re-experiencing; symptoms decreased with age. As in the model for overall symptoms, hypoconnectivity between the lMFG and lIFG was selected as a predictor of re-experiencing. Additionally, hypoconnectivity between the lMFG and right orbitofrontal cortex (rOFC) was associated with re-experiencing symptoms. Finally, hyperconnectivity between the right cingulate gyrus (rCG) and left parahippocampal gyrus (lPHG) was included in the model to predict avoidance.

Two additional predictors had average MIPs just below the 0.50 cutoff. Visual inspection of the MIP plots suggests that these predictors may also warrant consideration. Extraversion/surgency was near the cutoff as a unique predictor for avoidance, with higher extraversion related to lower avoidance. Connectivity between the rCG and lPHG, which was selected in the model for avoidance, was also close to the inclusion threshold for re-experiencing symptoms.

### Controlling for depression

The results for SSVS with depression included in the set of candidate predictors are shown in Table 4. Depression had an average MIP >0.99 for all outcomes. The average effect of depression ranged from *b* = 0.69 to *b* = 1.69 across outcomes and was the largest effect in each model. Comparing Tables 2 and 3, we see that many predictors were no longer included after accounting for depression symptoms. For example, when controlling for depression, poor sleep quality was only selected in the model to predict arousal. Conversely, positive urgency was included for all subscales when controlling for depression, but only for re-experiencing in the model without depression. The patterns of effects for lack of perseverance, orienting sensitivity, and nicotine dependence were most consistent across models with and without controlling for depression.

**Table 4:** Results from SSVS indicating predictors selected to model each PTSD score, controlling for depression.

**Selected predictor**	Outcome			
	**Overall**	**Re-experiencing**	**Avoidance**	**Arousal**
Self-report measures	MIP, Avg *B*	MIP, Avg *B*	MIP, Avg *B*	MIP, Avg *B*
Depression	0.99, 0.74	1.0, 0.69	1.0, 1.69	1.0, 1.37
Negative urgency			0.61, 0.36	
Lack of perseverance	0.93, −0.49			
Positive urgency		1.0, 0.51	0.50, 0.25	0.52, 0.21
Effortful control			0.63, 0.30	
Orienting sensitivity	0.48, 0.16^a^	0.55, 0.14	0.99, 0.59	0.81, 0.36
Nicotine dependence	0.80, 0.31	0.82, 0.24		
Gender (Ref = Male)			0.59, −0.24	
Poor sleep quality				0.53, 0.20
Brain connectivity measures				
L Superior temporal gyrus–R Orbitofrontal cortex	0.50, 0.31			
L Middle frontal gyrus–R Orbitofrontal cortex		0.56, −0.24		

*Note.* Results are averaged over 50 imputations. Predictors not selected for any outcome are omitted. Blank cells indicate predictors not selected for a given outcome. ^a^Coefficient was just below the MIP threshold of 0.5.

Of the brain FC measures, the only predictor that remained consistent when controlling for depression was connectivity between the lMFG and rOFC, and only in the model for re-experiencing. Hyperconnectivity between the left superior temporal gyrus (lSTG) and rOFC was selected as a predictor of overall PTSD symptom severity.

## Discussion

The present study is the first to investigate predictors of PTSD symptom dimensions using a powerful variable selection method applied across self-report and brain FC measures collected from a large sample of adults. We found that self-report measures and brain FC measures explained the unique variability in PTSD symptoms.

We found strong support for the roles of personality and impulsivity in predicting PTSD symptoms. Specifically, orienting sensitivity was the most consistent predictor across outcomes, with or without controlling for depression. Orienting sensitivity encompasses automatic attention to external and internal sensory events; this is related to general overreactivity to strong stimuli (Bolders et al. 2017). Temperament negative affect predicted re-experiencing and arousal, but this effect did not survive when controlling for depression symptoms. Surprisingly, in the model including depression symptoms, effortful control was a unique predictor positively related to avoidance. While high effortful control is generally protective against the overall development of PTSD, avoidance in particular is a strategic (i.e. effortful) process (Feuer et al. 2005).

Emotion-related impulsivity factors were included for all outcomes; notably, positive urgency was a strong and consistent unique predictor of re-experiencing, and negative urgency was a strong predictor for the other 3 PTSD outcome measures. Emotion-related impulsivity, in response to strong positive and negative emotions, is an important transdiagnostic risk factor for psychopathology (Johnson et al. 2013), and negative urgency in particular is a strong predictor of PTSD severity (Roley et al. 2017; Contractor et al. 2018). However, this pattern of effects differed when controlling for depression symptoms. Controlling for depression, positive urgency was also selected to predict avoidance and arousal symptoms, and negative urgency remained associated with avoidance symptoms only.

The finding that lack of perseverance was associated with lower overall symptom severity may seem counterintuitive; however, this finding replicates previous research in trauma-exposed individuals (Ceschi et al. 2014). Ceschi postulated that in the context of trauma, increased perseverance may have negative consequences for emotion regulation due to a greater tendency for rumination and less tendency to distraction.

Poor sleep quality predicted all PTSD symptom scales, but controlling for depression symptoms was only uniquely related to arousal. These findings support both the key role of sleep problems in depression and general PTSD symptoms, which may be mediated by rumination and other factors (Borders et al. 2015), as well as the unique physiological dimension of PTSD-related hyperarousal (Woodward et al. 2000).

Nicotine dependence was selected in the models for overall symptoms and re-experiencing, both with and without controlling for depression symptoms. A unique relationship between PTSD symptoms and nicotine dependence is well established, especially among men (Thorndike et al. 2006). Re-experiencing symptoms have been uniquely associated with both positive and negative reinforcement smoking motives, whereas overall symptom severity is associated only with negative reinforcement smoking motives (Mathew et al. 2015). In contrast, alcohol use, another common and maladaptive coping behavior, was not selected as a unique predictor of PTSD symptoms; this finding also supports the unique relationship between nicotine dependence and PTSD symptoms, especially re-experiencing, over and above the contribution of other symptom clusters and comorbidity (Breslau et al. 2003; Trautmann et al. 2015).

We also found gender differences for avoidance and arousal symptoms; the finding for avoidance symptoms remained when controlling for depression. Interestingly, although rates of PTSD are higher in women, even accounting for the differential risk of potentially traumatic events (Tolin and Foa 2006), we found lower predicted severity for women compared to men for these specific symptom dimensions—when controlling for impulsivity and personality dimensions.

In addition to self-report measures, results supported the importance of brain FC measures as unique predictors of PTSD symptoms. Connections among regions commonly ascribed to the DMN, CEN, and SN were selected for models predicting overall symptoms, re-experiencing, and avoidance. No connectivity measures were selected to predict arousal. Several brain FC measures were no longer included when controlling for depression.

Hypoconnectivity between the lMFG and lIFG was selected to predict both overall and re-experiencing symptoms. This is consistent with a recent review (Akiki et al. 2017) and metanalysis (Bao et al. 2021) showing that PTSD symptoms are associated with weakly connected CEN. Hypoconnectivity between the lMFG and rOFC was predictive of re-experiencing symptoms, and this specific relationship was the only brain connectivity predictor that was selected with or without controlling for depression. The rOFC is a region in the prefrontal cortex involved in cognitive processes and decision-making, and neurocircuitry models of PTSD emphasize the role of diminished prefrontal inhibitory control in PTSD (Rauch et al. 2006; Akiki et al. 2017). Conversely, hyperconnectivity between the rCG and lPHG was associated with avoidance symptoms in our sample and was narrowly excluded from the model for re-experiencing. This association of increased SN to DMN connectivity with PTSD symptoms has been noted in the literature and is consistent with neurocircuitry models of PTSD (Sripada et al. 2012; Akiki et al. 2017; Chen et al. 2021). Controlling for depression, overall symptoms were also predicted by hyperconnectivity of the lSTG and rOFC. Consistent with Bao et al’.s metanalysis (16), we found alterations in the left CEN that predicted PTSD symptoms.

Resting state fMRI data permits measures of FC, or statistical interdependencies of blood oxygen level-dependent (BOLD) signals between brain regions. Resting state FC reflects the strength of coupling between brain regions and is thought to reflect the history of co-activation among them. FC approaches can reveal the intrinsic or underlying functional architecture of the brain, and are complementary to task fMRI paradigms that reveal associations between BOLD signals and specific cognitive processes. Future directions should explore task fMRI datasets focusing on cognitive constructs implicated in PTSD, including threat, reward, and impulsivity.

Notably, when included as a candidate predictor, depression was the strongest predictor for all outcomes. Comorbidity in PTSD is prevalent and meaningful and has implications for PTSD severity, treatment, and course (Qassem et al. 2021). Researchers investigating psychiatric comorbidities and PTSD have found that comorbidity is not driven by symptom overlap for major depressive disorder (Franklin and Zimmerman 2001; Afzali et al. 2017) or sleep disorders (Spoormaker and Montgomery 2008). The inclusion of depression symptoms as a strong predictor of each measure of PTSD highlights the importance of continuing to investigate the important role of changes in cognition and mood in PTSD.

The measure of PTSD used for this study was developed according to DSM-IV criteria, and several changes were made to the diagnostic criteria for PTSD in the DSM-5. Among these changes, the diagnosis was moved from anxiety disorders to trauma, and the avoidance and numbing symptom cluster from DSM-IV was separated into separate symptom clusters for DSM-5; this change resulted in the requirement that a PTSD diagnosis include at least 1 avoidance symptom and also highlighted the role of negative alterations in cognitions and mood (Pai et al. 2017). The conceptualization of posttraumatic psychopathology continues to be refined and debated; several central issues include distinguishing predictors from core symptoms, how to conceptualize heterogeneity within the diagnosis, and categorical versus dimensional conceptualizations of trauma-related symptoms (Hawn et al. 2022).

Strengths of this study include the large adult sample, novel methodological approach, inclusion of self-report and brain connectivity measures, and examination of PTSD symptom dimensions. The study was cross-sectional, and we cannot establish the temporality of these associations, though theoretically, impulsivity and personality are stable characteristics in adulthood. An important future direction would be to investigate these associations in a prospective study, as well as how these associations change with time following the traumatic event. Another limitation is the use of a PTSD symptom measure developed according to DSM-IV criteria rather than the updated DSM-5 conceptualization of PTSD. However, we do not think these changes should substantially limit the generalizability of our results because our focus is on post-traumatic stress response dimensionally rather than diagnosis, and findings have shown that the symptom dimensions and diagnosis rates have not changed dramatically (Danzi and La Greca 2016; Cheng et al. 2021). Our analytical framework did not consider interaction effects, and in particular, the pattern of effects should be examined for sex differences (Pineles et al. 2017). Future research should also investigate how baseline psychological and neural characteristics interact with trauma exposure to predict PTSD outcomes.

## Author contributions

Sierra Bainter (Conceptualization, Formal analysis, Methodology, Writing—original draft, Writing—review & editing), Zachary Goodman (Data curation, Formal analysis, Visualization, Writing—review & editing), Lauren B. Kupis (Data curation, Formal analysis, Writing—review & editing), Kiara R. Timpano (Conceptualization, Writing—review & editing), and Lucina Uddin (Conceptualization, Supervision, Writing—review & editing).

## Funding

SAB received funding from grant K01-MH122805 from the National Institutes of Health. ZTG received funding from grant T32-HL007426 from the National Institutes of Health. LQU is supported by R21HD111805 and U01DA050987 from the National Institutes of Health. KRT received funding from grant R01MH110477 from the National Institutes of Health.

*Conflict of interest statement*: None declared.
